# Strain Analysis of GaN HEMTs on (111) Silicon with Two Transitional Al_x_Ga_1−x_N Layers

**DOI:** 10.3390/ma11101968

**Published:** 2018-10-13

**Authors:** Yuefei Cai, Chenqi Zhu, Ling Jiu, Yipin Gong, Xiang Yu, Jie Bai, Volkan Esendag, Tao Wang

**Affiliations:** Department of Electronic and Electrical Engineering, The University of Sheffield, Sheffield S1 3JD, UK; yuefei.cai@sheffield.ac.uk (Y.C.); czhu11@sheffield.ac.uk (C.Z.); ljiu1@sheffield.ac.uk (L.J.); y.gong@sheffield.ac.uk (Y.G.); x.yu@sheffield.ac.uk (X.Y.); j.bai@sheffield.ac.uk (J.B.); vesendag1@sheffield.ac.uk (V.E.)

**Keywords:** HEMTs, strain, Al_x_Ga_1−x_N, crack-free, silicon

## Abstract

We have designed and then grown a simple structure for high electron mobility transistors (HEMTs) on silicon, where as usual two transitional layers of Al_x_Ga_1−x_N (x = 0.35, x = 0.17) have been used in order to engineer the induced strain as a result of the large lattice mismatch and large thermal expansion coefficient difference between GaN and silicon. Detailed x-ray reciprocal space mapping (RSM) measurements have been taken in order to study the strain, along with cross-section scanning electron microscope (SEM) images and x-ray diffraction (XRD) curve measurements. It has been found that it is critical to achieve a crack-free GaN HEMT epi-wafer with high crystal quality by obtaining a high quality AlN buffer, and then tuning the proper thickness and aluminium composition of the two transitional Al_x_Ga_1−x_N layers. Finally, HEMTs with high performance that are fabricated on the epi-wafer have been demonstrated to confirm the success of our strain engineering and above analysis.

## 1. Introduction

In the last decade, III-nitride devices have been widely used in many applications, such as general illumination [1], radio-frequency communication [2] and power conversion [3], etc. Especially for electronic applications, GaN high electron mobility transistors (HEMTs) are expected to demonstrate a number of major advantages, such as a fast switching speed, low switching loss and high power conversion efficiency in comparison with silicon based counterparts [4]. Thus far, a number of substrates have been explored for the growth of GaN HEMTs, such as sapphire (Al_2_O_3_), silicon, silicon carbon (SiC) and free-standing GaN, among which silicon substrates are becoming more attractive to the semiconductor industry due to the mature silicon technology, good thermal conductivity and scalability. However, there is a large lattice mismatch and a large thermal expansion coefficient (TEC) difference between GaN and silicon, typically causing extensive cracks in the post-growth cooling down process [5,6,7,8,9], and thus posing a great challenge for growing GaN HEMTs on silicon substrates.

Until now, a number of approaches have been proposed, such as AlN/GaN super-lattice layers [10,11], Al_x_Ga_1−x_N-based interlayers [8,12,13,14], patterned silicon substrate [15,16] and step-graded Al_x_Ga_1−x_N strain-release layers [17,18,19,20]. The idea is to use the compressive strain that is built due to the GaN on these Al(Ga)N layers to compensate the tensile strain between GaN and silicon generated during the post-growth cooling down process. Among these methods, the graded Al_x_Ga_1−x_N method is very popular due to its easiness to achieve and analyze the strains in each layer. In detail, for the graded Al_x_Ga_1−x_N buffer layers, a large number (>4) of Al_x_Ga_1−x_N layers [21] and different Al compositions (from 0.75 to 0.25) have been reported [22]. However, the growth procedures are quite time-consuming, leading to a high manufacturing cost [12]. On the other side, due to the complex epi-structure design, the strain-released mechanism is still not explicitly explained yet.

In this paper, we design a very simple HEMT epi-structure with one AlN buffer layer, only two graded transitional Al_x_Ga_1−x_N layers and then a GaN/Al_0.2_Ga_0.8_N heterostructure. This simple structure allows us to analyze the strain more clearly for the graded Al_x_Ga_1−x_N transitional layers with different compositions and thicknesses. By measuring X-ray reciprocal space mapping (RSM), the strain components in our Al_x_Ga_1−x_N layers can be obtained and then feed back into epi-wafer growth. Finally, the HEMTs with high performance have been demonstrated, verifying the quality of our GaN HEMT epi-wafers.

## 2. Materials and Methods

**Figure 1:**
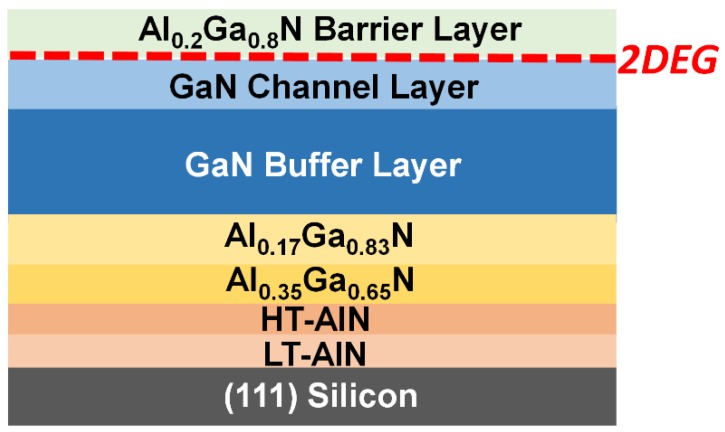
Epi-structure designed for our GaN high electron mobility transistors (HEMTs) with two graded Al_x_Ga_1−x_N strain-compensation transitional layers grown on (111) silicon.

Figure 1 schematically displays the GaN HEMTs epi-structure used. First, a standard 2-inch (111) silicon wafer is loaded into a low-pressure metalorganic vapour-phase epitaxy (MOVPE) system (Aixtron, Herzogenrath, Germany) and subjected to a high temperature (1320 °C) annealing process under H_2_ ambiance to remove any contaminants and native oxides. Subsequently, the temperature is decreased to 1000 °C and a trimethylaluminium (TMA) pre-flow is conducted without any NH_3_ flowing for 40 s. A thin low-temperature AlN (LT-AlN) layer is then grown, followed by a high temperature AlN (HT-AlN) layer grown at 1297 °C. The thickness of the AlN layer is 260 nm in total. Next, two layers of Al_0.35_Ga_0.65_N and Al_0.17_Ga_0.83_N have been further grown as strain-compensation transitional layers. After finishing the growth of the two Al_x_Ga_1−x_N transitional layers, a 1.2 µm (0001) GaN layer was then grown, followed by a final 25 nm Al_0.2_Ga_0.8_N barrier layer. During the cooling down procedure, N_2_ and NH_3_ are used as cooling gases in order to eliminate any micro cracks generated on the final Al_x_Ga_1−x_N layer which has been accepted as a result of H_2_ enhanced surface etching [23].

## 3. Results and Discussions

**Figure 2:**
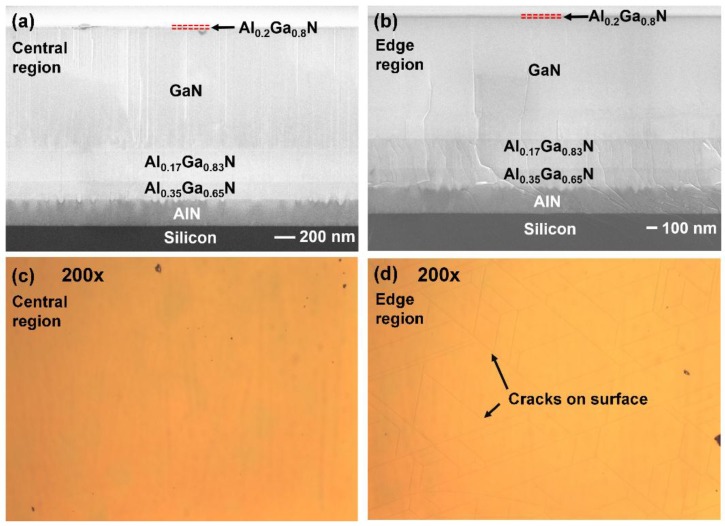
Cross-sectional SEM images of the epi-structure of GaN HEMT on silicon (**a**) in the central region and (**b**) in the edge region; Optical microscope images of the wafer surface (**c**) in the central region and (**d**) in the edge region.

The whole wafer has been examined across two inches by optical microscopy, confirming that it is crack-free except for the edge region of the wafer, as shown in Figure 2c,d. Furthermore, cross-sectional scanning electron microscope (SEM, Raith, Dortmund, Germany) measurements have been taken as shown in Figure 2a,b, taken from the central part and the edge part, respectively, indicating that the thicknesses for the AlN buffer layer, the Al_0.35_Ga_0.65_N layer, the Al_0.17_Ga_0.83_N layer and the GaN layer are 258 nm, 180 nm, 290 nm and 1.18 µm, respectively.

By comparing Figure 2a,b, the AlN layer in the central part is flat and crack-free, which eventually leads to a crack-free region for the final device structure. In contrast, at the edge region, most cracks generated in the AlN layers merge into the second Al_x_Ga_1−x_N layer, thus filtered by the Al_x_Ga_1−x_N layers. However, there are still a few cracks penetrating the GaN layer which extend to the surface, as shown in Figure 2d. The differences of the crack densities in the AlN layers between the central and the edge regions may be caused by the differences in wafer bowing, which are measured to be 121 m and 63 m for the wafer centre and wafer edge, respectively. At the same time, we also note that our wafer centre bowing is comparable and even smaller than the reported 119 m in [18].

**Figure 3:**
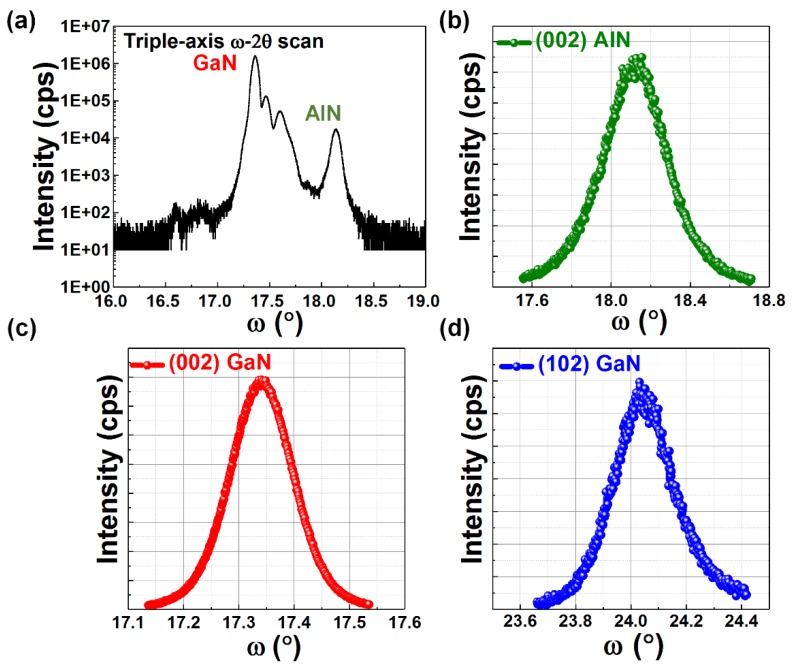
XRD results: (**a**) ω-2θ scan aligned on AlN and XRD rocking curves of (**b**) (002) AlN, (**c**) (002) GaN and (**d**) (102) GaN.

The crystal quality has been further characterized by X-ray diffraction (XRD, Bruker, Billerica, MA, USA) measurements as shown in Figure 3b–d, demonstrating that the full-width half-maximum (FWHM) values for the AlN and the GaN layers measured across the (002) reflection are 0.3783° and 0.1348°, respectively. The FWHM value for the GaN layer measured across the (102) GaN reflection is 0.2533°. Our (002) direction XRD result is better than the reported 0.294° and 0.2° in References [18,19], and close to the reported 0.122° and 0.132° in References [12,22]. Moreover, our (102) GaN direction XRD result is also comparable to the reported 0.24° in Reference [19]. This represents that a high crystal quality has been achieved by our method, and also implies that it is crucial to obtain an AlN buffer with high quality, which is one of the factors leading to our high quality GaN grown on top.

According to Reference [24], the screw dislocation density ($D_{screw}$) and edge dislocation density $(D_{edge}$) in the GaN layer can be calculated by using the below equations:

(1) $$D_{screw}=\frac{\beta_{\left(0002\right)}^{2}}{4.35b_{screw}^{2}}$$

(2) $$D_{edge}=\frac{\beta_{\left(10\bar{1}2\right)}^{2}-\beta_{\left(0002\right)}^{2}}{4.35b_{edge}^{2}}$$

where, $\beta_{\left(0002\right)}$ and $\beta_{\left(10\bar{1}2\right)}$ are the FWHM of symmetric (0002) and asymmetric (10$\bar{1}$2) ω scan. Burgers vector lengths for screw-type and edge-type are 0.5185 nm $\left(b_{screw}\right)$ and 0.3189 nm $\left(b_{edge}\right)$, respectively.

Thus, we can get

$$D_{screw}=\frac{\beta_{\left(0002\right)}^{2}}{4.35{\left(0.5185\;\mathrm{nm}\right)}^{2}}=4.733\times 10^{8}\;{\mathrm{cm}}^{-2}$$

$$D_{edge}=\frac{\beta_{\left(10\bar{1}2\right)}^{2}-\beta_{\left(0002\right)}^{2}}{4.35{\left(0.3189\;\mathrm{nm}\right)}^{2}}=3.167\times 10^{9}\;{\mathrm{cm}}^{-2}$$

With our growth method, the edge dislocation density is almost 7.3 times the screw dislocation density, thus edge dislocation dominates all the dislocations. In future, we will focus on reducing the edge dislocation to further optimize the GaN buffer layers.

**Figure 4:**
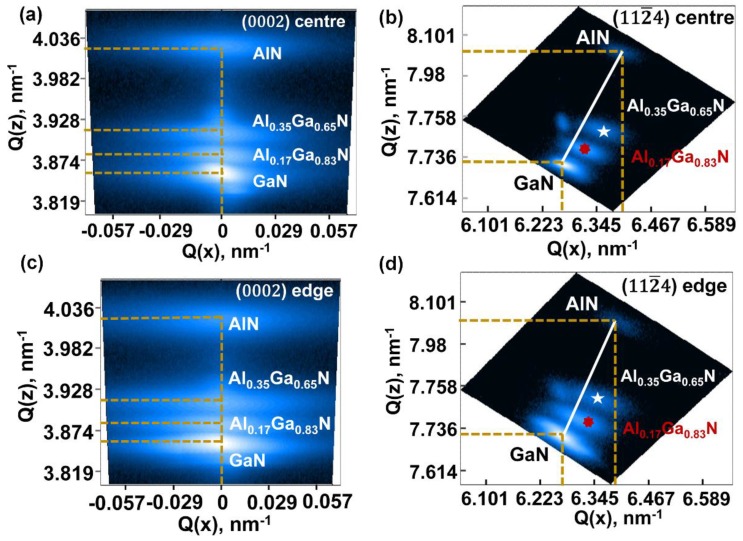
RSM results: (**a**) (0002) plane, (**b**) (11$\bar{2}$4) plane for central region, (**c**) (0002) plane and (**d**) (11$\bar{2}$4) plane for edge region.

To further analyze the strain and Al composition effects on the strain compensation layers separately [25], reciprocal space mapping (RSM, Bruker, Billerica, MA, USA) measurements have been taken from the central part and the edge part along the [0002] and [11$\bar{2}$4] directions, which are shown in Figure 4. The golden dash line indicates the coordinates of GaN, Al_x_Ga_1−x_N and AlN layers in RSM maps. The solid white line shows the fully relaxed Al_x_Ga_1−x_N layers grown AlN. In Figure 4a,c, in the (0002) Bragg reflection plane, three epitaxial layers overlap with each other at Q_x_ = 0, making it difficult to investigate the in-plane strain distribution of Al_x_Ga_1−x_N transitional layers. We rotated certain angles to the (11$\bar{2}$4) Bragg reflection plane to better analyze the in-plane strain, as shown in Figure 4b,d.

Based on the RSM measurements, the compressive in-plane strain components accumulated in each Al_x_Ga_1−x_N layer have been calculated according to the below equations:

(3) $$a=\sqrt{\frac{4\left(h^{2}+hk+k^{2}\right)}{3q_{x}^{2}}}$$

(4) $$c=\frac{l}{q_{z}}$$

(5) $$\tan\left[\alpha \left(x\right)\right]=\frac{q_{x}-q\left(x_{0}\right)}{q_{z}-q\left(z_{0}\right)}$$

(6) $$\varepsilon_{zz}=-D\left(x\right)\varepsilon_{xx}$$

(7) $$D\left(x\right)=2\frac{C_{13}\left(x\right)}{C_{33}\left(x\right)}$$

where (*q_x_*,*q_z_*) is the coordinates in the map, corresponding to the latticed constants (a,c); (h,k,l) is the Bragg reflection direction; (*q*($x_{0}$),*q*($z_{0}$)) is the coordinates of the fully relaxed reciprocal lattice points (RLPs) with the same Al composition as the (*q_x_*,*q_z_*); α(x) is the angle between the Q_z_ axis and the extended line interpolated from the two points described above; $\varepsilon_{zz}=\frac{\left[c-c_{0}\left(x\right)\right]}{c_{0}\left(x\right)},\varepsilon_{xx}=\frac{\left[a-a_{0}\left(x\right)\right]}{a_{0}\left(x\right)}$ are in-plane and out-plane strain components, respectively, among which c and a are measured lattice parameters, where c_0_ and a_0_ are the relaxed parameters from Vegard’s law; C_i,j_(x) are the elastic constants.

For the GaN and AlN lattice constant, a_GaN_ = 3.189 Å, c_GaN_ = 5.185 Å, a_AlN_ = 3.112 Å, c_AlN_ = 4.982 Å are used. For the elastic constants, C_13_ = 103 GPa, C_33_ = 405 GPa for GaN [24] and C_13_ = 108 GPa, C_33_ = 373 GPa for AlN [26] are used for calculation. We first calculate initial D(x) values for the first and second Al_x_Ga_1−x_N layer, then use these initial D(x) values to obtain an initial α(x) value and Al composition value. After several steps of iterative operations using the least-square method for error-minimization, we can finally get accurate values of the in-plane strain component $\varepsilon_{xx}$, as listed in Table 1.

From Table 1, we can see that, the strain components in the wafer central region are larger than the one in wafer edge region. According to Reference [12], there are three strains causing the cracks formation. Namely, the lattice mismatched strain, the grain size growth strain (or dislocation relaxation-related strain) and the thermal expansion coefficient mismatched strain. For the former two strains, they exist during the growth. For the thermal strain, it occurs during the cooling down procedure. At the growth temperature, initial tensile strain accumulates during AlN growth. After Al_x_Ga_1−x_N growth, compressive strain accumulated. Finally, during the GaN growth, compressive strain increases at first then decreases due to the dislocation relaxation. Given that the GaN thicknesses and dislocation densities are almost the same for the wafer centre and edge, an equal amount of compressive strain accumulated in GaN layers and was consumed by dislocations during the growth procedure for wafer centre and edge. However, cracks form only at the wafer edge after cooling down, so we can conclude that not enough initial compressive strain accumulated during the Al_x_Ga_1−x_N transitional layers for the wafer edge leads to the cracks formed at the edge region when cooling down to room temperature.

To evaluate the whole HEMT epi quality, Hall measurements have also been conducted. The Ti/Al/Ti/Au (20/150/50/80 nm) alloys have been deposited as pads and thermally annealed in order to form Ohmic contacts. The Hall result shows that a carrier mobility of the two-dimensional electron gas (2DEG) is as high as 1600 cm^2^ V^−1^ S^−1^, which is less than, but still comparable to the reported 2150 cm^2^ V^−1^ S^−1^ in Reference [12]. The high carrier mobility confirms the success of the strain compensation and a low dislocation density in the GaN channel layer, otherwise carrier scattering is expected to lead to a low mobility.

To further verify the epi quality, GaN HEMT devices are also fabricated on the HEMT epi-wafer. The fabrication process is described as below: A 300 nm depth mesa is etched down to define the active region for the HEMT by inductive coupled plasma (ICP) etching, then the metal stack of Ti/Al/Ti/Au (20/150/50/80 nm) is deposited and then annealed under a 850 °C N_2_ ambient atmosphere for 30 s in order to form Ohmic contacts for the source and drain of the HEMT. Finally, a Ni/Au (50/150 nm) alloy is deposited in order to form a Schottky gate for the HEMT.

**Figure 5:**
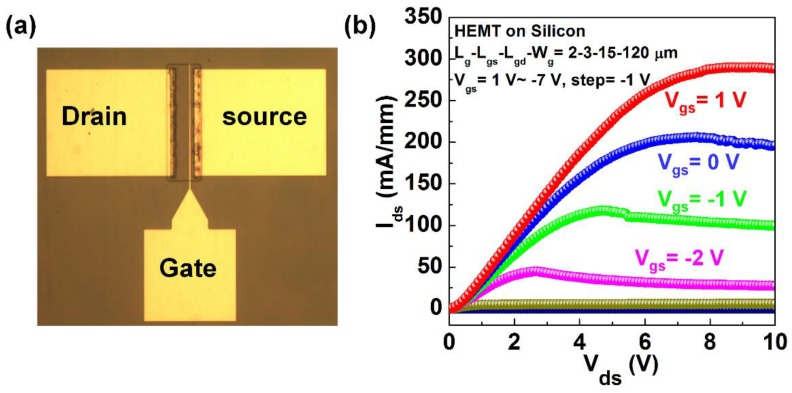
A schematic configuration of the HEMT device (**a**); current–voltage (I-V) as a function of gate voltage (**b**).

Figure 5a shows a schematic configuration of the HEMT device. Figure 5b shows a typical current-voltage (I-V) as a function of gate voltage, measured by a two-channel Keithley 2612B source meter (Cleveland, OH, USA). For the HEMT device with a gate length, gate-to-drain distance, gate-to-source distance and gate width of 2 µm, 3 µm, 15 µm, 120 µm, respectively, it shows a maximum current density of 290 mA/mm at V_gs_ = 1 V and V_ds_ = 8.8 V and specific-on resistance of 0.427 Ω·mm^2^, comparable to the reported value of 688 mA/mm at V_ds_ = 9 V in Reference [12], considering the large active area of our HEMT. These results indicate a high mobility and carrier density of the channel layers of our HEMT epi.

## 4. Conclusions

To conclude, we have achieved a crack-free GaN HEMT epi-wafer grown on silicon by properly tuning two Al_x_Ga_1−x_N transitional layers. The compressive strain of GaN on the Al_0.35_Ga_0.65_N and Al_0.17_Ga_0.83_N layers on the AlN buffer is good enough to compensate the tensile strain between GaN and silicon. As a result of a high crystal quality AlN buffer layer, high quality GaN HEMTs on silicon with a mobility of 1600 cm^2^ V^−1^ S^−1^ and a current density of 290 mA/mm for 120 µm-gate HEMTs have been achieved.

## Figures and Tables

**Table 1:** Calculated in-plane strain components of the two strain-compensation Al_x_Ga_1−x_N layers for both the wafer central and edge regions measured along the [11$\bar{2}$4] direction.

Location	1st layer Al_0.35_Ga_0.65_N	2nd layer Al_0.17_Ga_0.83_N
central	−0.00227	−0.00189
edge	−0.00174	−0.0011
